# Natural Vitamins and Novel Synthetic Antioxidants Targeting Mitochondria in Cognitive Health: A Scoping Review of In Vivo Evidence

**DOI:** 10.3390/antiox15010078

**Published:** 2026-01-07

**Authors:** Alexia Squillace, Malika G. Fernando, Kirstin Sullivan, Hosen Kiat, Ralph N. Martins

**Affiliations:** 1 Faculty of Medicine, Health and Human Sciences, Macquarie University, Sydney, NSW 2109, Australia; alexiasquillace@gmail.com (A.S.); kirstin.t.sullivan@gmail.com (K.S.); hosen.kiat@chi.sydney (H.K.); ralph.martins@mq.edu.au (R.N.M.); 2 College of Health and Medicine, Australian National University, Canberra, ACT 2601, Australia

**Keywords:** Alzheimer’s disease, dementia, cognitive impairment, in vivo animal models, mitochondria-targeted antioxidants, oxidative stress

## Abstract

Mitochondrial dysfunction and oxidative stress are crucial contributors to the pathogenesis of Alzheimer’s disease (AD) and dementia exhibiting cognitive decline at the early stage of neurodegeneration. Natural vitamin antioxidants (NVAs) and novel mitochondria-targeted antioxidants (MTAs) are proposed as potential therapeutics though conclusive evidence is lacking. Objectives were to examine in vivo evidence on NVAs and MTAs for preventing and/or treating cognitive decline leading to dementia, to identify the most promising antioxidants, and highlight translational gaps. Methods followed PRISMA-ScR guidelines. MEDLINE, EMBASE and Scopus were searched for English language in vivo experiments assessing NVAs or MTAs in AD and dementia. A total of 25 studies (13 NVAs; 12 MTAs) met inclusion criteria. NVAs (Vitamin A, B, C, E) demonstrated mixed efficacy in reducing oxidative stress and improving cognitive outcomes, with Vitamin E showing the most consistent neuroprotective effects. MTAs (MitoQ, MitoTEMPO, SS31, SkQ1) improved mitochondrial dynamics and cognitive performance and reduced dementia-related pathology. Both NVAs and MTAs improved biomarker profiles and cognitive outcomes in vivo animal models of AD and dementia, but MTAs showed more robust and consistent efficacy by directly targeting mitochondrial pathways. Given the favourable safety profiles of MTAs in other clinical conditions, early-phase human trials in dementia and AD are warranted to evaluate their long-term cognitive benefits.

## 1. Introduction

Cognitive decline leading to dementia is a major global health concern with significant morbidity and mortality internationally, and no successful curative treatments [1,2,3]. Mild cognitive impairment (MCI) affects up to one-fifth of adults over 65 years old and substantially increases the risk of progression to dementia [4]. Although the pathogenesis of dementia is not completely understood, mitochondrial dysfunction through induction of oxidative stress and deposition of pathogenic proteins has been recognised as an early and central driver of neurodegeneration [5,6,7,8].

Mitochondria are essential for adenosine triphosphate (ATP) production through oxidative phosphorylation, cell metabolism, homeostasis and survival, and are regulated by genes and transcription factors (TF) encoded by mitochondrial and nuclear DNA through mitochondrial biogenesis [9]. Mitochondria are particularly susceptible to oxidative stress; dysfunction results in excess reactive oxygen species (ROS), which can induce mitochondrial DNA mutations and disrupt neuronal synaptic function [5,7,10]. This creates a vicious cycle whereby damaged mitochondria generate more ROS, exacerbating mitochondrial damage, leading to further ROS accumulation. In conjunction, accumulation of amyloid-β (Aβ) and hyperphosphorylated tau (p-tau) in AD and dementia exacerbates mitochondrial dysfunction, promoting mitochondrial apoptosis and necrosis, thereby further perpetuating neuroinflammation and ROS generation [5,7,8]. This cascade leads to synaptic degeneration and neuronal loss, contributing to progressive brain atrophy due to the limited regenerative capacity of the central nervous system [5,6,7,8].

As mitochondrial dysfunction and oxidative damage represent crucial and early mechanisms in the pathogenesis of dementia, particularly AD, there is growing interest in novel therapeutic approaches targeting these processes [5,7,8]. Natural vitamin antioxidants (NVAs) such as Vitamins E, C, Bs and A have been trialled in animal models and human studies of cognitive decline leading to dementia. However, their neuroprotective efficacy in human trials remains mixed and inconclusive [11,12]. Consequently, synthetic mitochondria-targeted antioxidants (MTAs) have been developed to selectively accumulate within mitochondria by targeting specific components of the mitochondrial membrane (MM), thereby reducing off-target metabolism and enhancing antioxidant efficacy [6,9,13]. Several MTAs (including MitoQ, SkQ1, MitoTEMPO, and SS31) have shown favourable neuroprotective effects in vivo, predominantly in animal models of AD, and some have also been explored in human studies (mainly in non-cognitive indications), such as MitoQ for vascular function [14] and SkQ1 is commercially available for the treatment of dry eye syndrome [15], suggesting acceptable safety profiles. However, there are very limited studies testing their effect on cognition and/or dementia in human trials.

This scoping review aims to systematically summarise the current in vivo evidence of both natural vitamins with antioxidant effects and novel synthetic MTAs in mitigating cognitive decline, as well as to highlight particularly promising MTAs and identify any research gaps. This research will serve to inform the design needed for human randomised-control trials using MTAs in various populations at risk of or diagnosed with dementia or other neurocognitive disorders. To our knowledge, this is the first review to comprehensively synthesise in vivo evidence on both NVAs and MTAs in the context of cognitive decline.

## 2. Materials and Methods

### 2.1. Type of Study

This scoping review is based on the Preferred Reporting Items for Systematic Reviews and Meta-analysis Protocols for Scoping Reviews (PRISMA-Scr) guidelines.

### 2.2. Review Question

The review question was formulated using the PICO framework as follows.

• Population: In vivo models of AD, dementia, cognitive decline, ageing, oxidative stress or mitochondrial dysfunction.
• Intervention: Natural vitamin antioxidants and mitochondria-targeted antioxidants.
• Comparison: Control or usual care.
• Outcome: Cognition, inflammation, mitochondrial function or other cognitive health markers.

### 2.3. Eligibility Criteria

Studies from any timeframe were included based on the following criteria:

• Inclusion criteria: English language, in vivo, specified antioxidants, outcome: dementia, cognition, cognitive or mitochondrial health.
• Exclusion criteria: Non-English language, non in vivo, multivitamin supplementations or vitamin precursors, irrelevant outcomes.

### 2.4. Information Sources and Search Strategy

Databases searched included MEDLINE, EMBASE and Scopus. The strategy was developed around the research question and refined by an experienced librarian. The final EMBASE strategy is detailed in Supplementary Materials Table S1. Additional sources of evidence were obtained through hand searching. The results were imported into Covidence, where duplicates were automatically removed.

### 2.5. Selection of Sources of Evidence

Titles and abstracts were screened independently by two blinded reviewers. Full texts were assessed by a single reviewer, with conflicts resolved by a third reviewer.

### 2.6. Data Charting Process and Items

Data were extracted into Microsoft Excel based on pre-defined study characteristics and outcomes, as agreed upon by three reviewers.

### 2.7. Synthesis of Results

A qualitative synthesis of results was performed, summarising each treatment and their effects on relevant parameters alongside rigorous analysis of included studies.

### 2.8. Patients and Participants Involvement

Patients and/or the participants were not involved in the design, or conduct, or reporting or dissemination plans of this research.

## 3. Results

**Figure 1:**
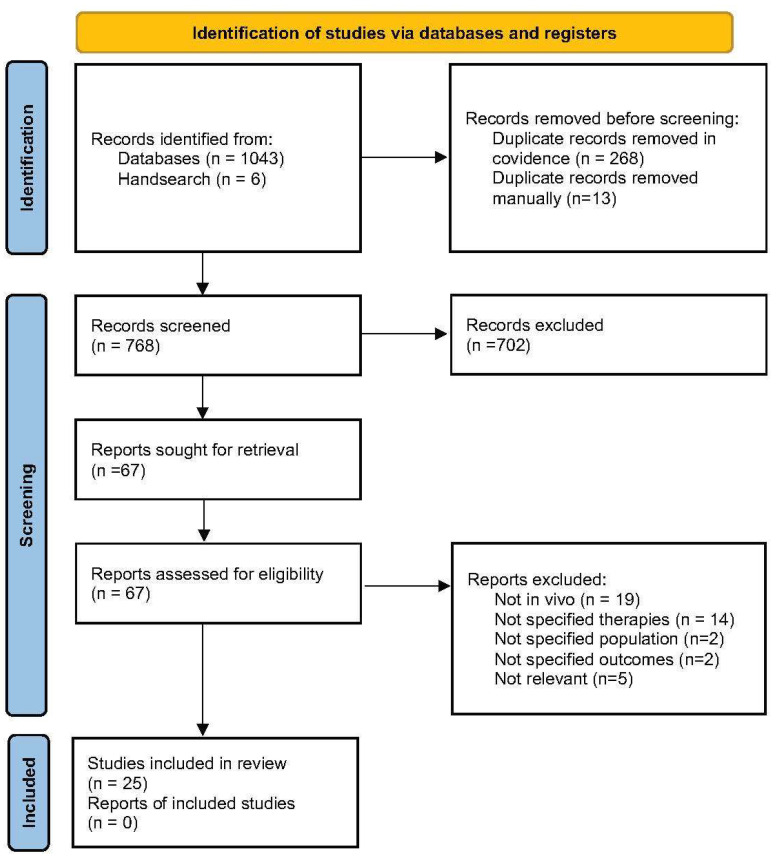
PRISMA flowchart showing the process of identification, title and abstract screening, full text screening and final included studies.

Of the 768 articles identified, 25 studies (13 NVAs; 12 MTAs) were included for analysis (Figure 1). Study characteristics are presented in Table 1, with key findings discussed below. The full EMBASE search strategy and PRISMA-ScR checklist are provided in the online Supplementary Materials.

### 3.1. Natural Vitamin Antioxidants

#### 3.1.1. Vitamin E

In the present review, Vitamin E (Vit.E) was the most extensively studied NVA, examined across four in vivo studies (including one study in mice, one in flies, one evaluating Trolox in mice and one assessing α-tocopherol on rats), and all demonstrated positive neuroprotective outcomes. One study compared α-tocopherol therapy, a potent form of Vit.E, in wild-type (WT) wistar rats vs. the OXYS rat model, which displays accelerated ageing and increased susceptibility to oxidative stress. They found improvement in oxidative stress markers, such as superoxide dismutase and lipid peroxidation, along with improvement of short-term memory to the level of age-matched controls [25]. In another study, the APP/PS1 mouse model, which harbours the *Amyloid Precursor Protein* (Swedish Mutation) (*APPswe*) and the *Presenilin 1* *(Psen1)* gene mutations linked to familial AD, was treated with Trolox, a synthetic water-soluble analogue of α-tocopherol [26,41]. Trolox treatment improved markers of oxidation in the acute (24 h) treatment group but failed to reduce these markers significantly in the chronic (15 days) treatment group. However, the chronic group exhibited improved neurite morphology, suggesting that antioxidant therapy can reverse neuronal damage in AD. Another study exposed WT C57BL/6J mice to a pollutant known to cause cognitive impairment and toxicity, PM2.5, followed by administration of Vit.E. Exposure to PM2.5 caused increased brain Aβ, glial activation, oxidative stress and cognitive decline, all of which Vit.E was able to reverse [27]. Finally, a study comparing Vit.E with Vitamin C treatment in Tau-expressing *Drosophila melanogaster* flies found that both vitamins reduced oxidative stress, partially restored normal larvae activity, with Vit.E also modestly improving adult fly motor activity. Remarkably, these changes occurred without alteration in tau phosphorylation, suggesting Vit.E’s benefit was primarily through its antioxidant effect [28].

#### 3.1.2. Retinoic Acid

Both studies identified in this review investigated downstream metabolites of vitamin A, which exert indirect effects on oxidative stress and mitochondrial function via gene regulatory mechanisms [42]. The first study found that retinoic acid (RA) treatment in aged WT mice (C57BL/6 Jico) ameliorated the reduced long-term potentiation (LTP) and impaired cognition, with mice outperforming aged-matched controls in hippocampal memory testing [23]. This was inhibited by co-administration of the RA receptor (RAR) antagonist. These findings were attributed to the increase in *RAR* gene transcription, an important transcription factor (TF) for many genes related to synaptic plasticity, neurotropism and cholinergic proteins [23]. Another study used All-trans retinoic acid (ATRA) in the 3xTg mouse model of AD, which harbours 3 gene mutations linked to familial AD (*APPswe*, *Psen1M146V* and *MaptP301L*), finding that the decreased neural stem cell proliferation (NSC) and neuroinflammation induced by AD could be ameliorated by ATRA supplementation [24]. This was attributed to its anti-inflammatory actions and regulation of gene expression responsible for NSC differentiation, cell cycle regulation and microglial activation.

#### 3.1.3. Methylcobalamin (Vitamin B12)

A study supplementing healthy 6 wk old mice (C57BL/6) exposed to the pollutant PM2.5 with methylcobalamin, a Vitamin B12 metabolite with neuroprotective and antioxidant effects, demonstrated restored cognitive ability in a spatial memory test. This was attributable to the reduced mitochondrial-associated neuronal apoptosis observed and decreased markers of ROS in mice brain tissue [19].

#### 3.1.4. Folate (Vitamin B9)

In one study, folate was injected directly into aged (21 mo) WT mice (C57BL/6) dentate gyri, resulting in improved locomotor activity, reduced anxiety–depression-like behaviour and improved long-term memory. These results were attributed to folate’s ability to modulate DNA methylation and interact with the TF Folate Receptor α to promote transcription of genes related to cell rejuvenation [17]. A study on Adult male wistar rats induced to exhibit AD-like pathology by homocysteine (Hcy) infusion found that folic acid (FA) was able to reduce plasma Hcy and decrease Aβ and p-tau burden and synthesis in hippocampal neurons [18]. Hcy has a role in disturbing DNA methylation, causing upregulation of AD-related genes, as well as inducing oxidative stress. Therefore, FA’s effect in this study may be attributed to its role in DNA methylation and anti-Hcy effect, causing a downstream reduction in oxidative stress [18].

#### 3.1.5. Niacin (Vitamin B3)

Two studies investigating the Vitamin B3 derivative nicotinamide riboside (NR) on mouse AD models were included in this review [20,21]. One study utilised the 3xPB mouse model (3xTg *APP* mutated mice heterozygous for Polβ), which has been shown to exhibit more similarities with the human AD transcriptome than 3xTg models alone. 3xPB mice were supplemented with NR from 16 to 18 mo old and their brain ROS interactome and proteome were analysed, finding reduced markers of oxidative stress, reduced neuronal apoptosis and increased ATP. Interestingly, despite dysregulation of the proteome in AD mice, there were only discrete changes in the proteome after NR treatment related to lipid metabolism, radical scavenging and neuronal density [21]. A similar study on NR treatment utilised the Tg2576 mouse model of AD, which overexpresses *APPswe*, crossed with PGC-1α^−/−^ mice, to examine the PGC-1a (peroxisome receptor-gamma co-activator 1) and BACE1 (β-secretase) pathway, which is responsible for the production of Aβ [43]. NR treatment for 6 months initiated at 5 mo. of age was found to enhance PGC-1a function, leading to a range of effects including increased BACE1 degradation and resultant decreased Aβ levels and regulation of mitochondrial gene expression, ultimately leading to improved hippocampal memory in mice [20].

#### 3.1.6. Thiamine (Vitamin B1)

One study was identified using a synthetic derivative of thiamine, benfotiamine, in a the P301S TG mouse model of tauopathy, which is known to cause frontotemporal dementia via overexpression of the *MaptP301S* gene mutation. Mice were treated with benfotiamine from 1 to 10 mo. old, and were found to have a longer lifespan, better cognitive outcomes, reduced advanced-glycation end products, reduced oxidative stress and preserved motor neurons in the spinal cord. These results were be attributed to the reduction in tau pathology and neurofibrillary tangles, and improved mitochondrial antioxidant enzymes and biogenesis. The study hypothesised that these outcomes may be due to benfotiamine’s role in transcription of the nuclear factor erythroid 2-related factor 2/antioxidant response element-driven pathway (Nrf2/ARE pathway), which regulates mitochondrial function, oxidation and inflammation, and is a crucial defence mechanism against cellular damage and metabolic dysfunction. However, intriguingly, Nrf2-dependent gene expression was only increased in WT mice, not the diseased mouse model [16].

#### 3.1.7. Pantetheine (Vitamin B5)

One study was identified in this review investigating a vitamin B5 metabolite, pantetheine, and its role in the 5XFAD mouse model of AD, which expresses five mutations of familial-AD-associated genes to cause Aβ plaque deposition and gliosis [22]. Pantetheine treatment was observed to decrease Aβ plaque burden, astrogliosis and microgliosis, which corresponded to a decrease in aggressive behaviour. This was attributed to an upregulation in genes that are suppressed and downregulation of genes that are activated in the 5XFAD mouse model of AD, many of which are involved with gliosis, ROS and Aβ formation [22].

### 3.2. Mitochondria-Targeted Antioxidants

**Figure 2:**
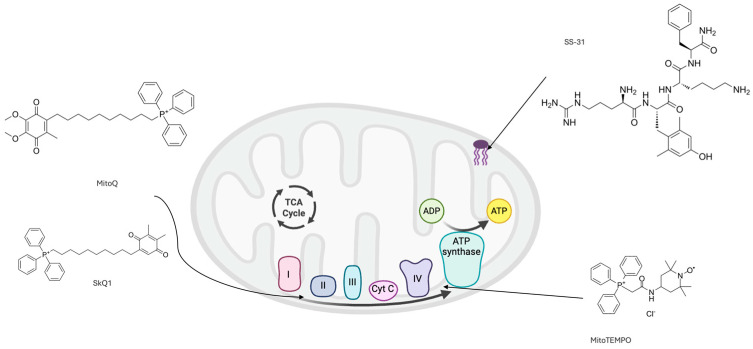
Mitochondria-specific targets of MTAs. MitoQ and SkQ1 accumulate in the lipid bilayer to shuttle electrons between the electron transport chain complexes (I = NADH dehydrogenase, II = succinate dehydrogenase, III = cytochrome bc_1_ complex, and IV = cytochrome c oxidase). MitoTEMPO similarly accumulates in this lipid bilayer to exert antioxidant effects. SS-31 targets cardiolipin in the inner mitochondrial membrane to exert antioxidant effects.

The mechanisms of action of the included MTAs are presented in Table 2, and the mitochondria-specific targets of MTAs are depicted in Figure 2.

#### 3.2.1. MitoQ

In the 3xTg AD mouse model, MitoQ treatment reduced synaptic loss, astrogliosis, markers of tau metabolism and Aβ accumulation in mice cortex [29]. This was reflected in vivo, showing improvement of spatial cognitive performance to the level of WT mice [29]. In a *C. elegans* AD model, MitoQ extended lifespan and healthspan, indicated by delayed paralysis onset. This effect was linked to protection of electron transport chain (ETC) complexes I and IV, despite no reduction in oxidative stress, mitochondrial DNA damage or energy metabolism [30].

#### 3.2.2. MitoTEMPO

MitoTEMPO treatment was studied in a WT mouse model (C57BL/6J) of hypoglycaemia-induced cognitive impairment, resulting in reduced hippocampal and cortical neuronal cell damage, oxidative stress, blood–brain barrier (BBB) permeability, and markers of mitochondrial dysfunction while improving pericyte cell number and function. These cellular effects contributed to the improvement in cognition seen in the MitoTEMPO group [31].

#### 3.2.3. SS31

Five studies investigating SS31 (Szeto-Schiler), or Elamipretide, in mouse models of AD and/or cognitive impairment were included in this review, all of which demonstrated favourable neuroprotective outcomes. The earliest study identified (2016) explored the use of SS31 in the senescence-accelerated mouse-prone 8 (SAMP8) mouse model of ageing, which develop AD pathological markers such as Aβ deposits and tau hyperphosphorylation, synaptic loss and subsequent reduced cognition [36]. They reported treatment with SS31 preserved mitochondrial structure and key synaptic proteins, improved parameters of mitochondrial function and lowered hippocampal Aβ [36]. These cellular improvements were reflected in the improved spatial learning and memory results in SS31 treated mice compared to untreated controls. A later study led by the same group (Jia et al.) investigated the APP/PS1 AD mice model treated with SS31 and found reduced Aβ plaque area and concentration in the hippocampus, reduced ROS in the hippocampus, partially restored changes to mitochondrial fusion/fission proteins, restored synaptic protein expression and inhibited neuronal apoptosis in the hippocampus [39]. These results were reflected by improved episodic and non-spatial memory and behavioural outcomes in SS31 treated mice compared to controls, restoring memory to similar levels of WT mice.

Another study on the effects of SS31 on an in vivo APP Tg2576 mouse model of AD revealed altered expression of mRNA and proteins involved with mitochondrial dynamics, biogenesis and synaptic regulation to a more favourable profile than controls, resulting in improved mitochondrial function and reduced cortical Aβ burden [37]. In addition, another study on APP/PS1 AD mice exhibited in real time that Aβ plaque induces mitochondrial oxidative stress in mice cortex using multiphoton microscopy [40]. SS31 was found to reduce this oxidative stress, notably showing a more robust effect in female mice. They also reported that SS31 reduced Aβ plaque-associated neurotoxicity and decreased dystrophic neurites, without altering Aβ plaque burden. Finally, an alternate mouse model of lipopolysaccharide-induced cognitive impairment found that SS31 also reduced oxidative stress, inflammation, mitochondrial dysfunction and neuronal apoptosis, upregulated pathways involved in neuronal synaptic function and increased hippocampal dendritic spine density. This resulted in enhanced hippocampal cognition including spatial memory and contextual fear conditioning [38].

#### 3.2.4. SkQ1

Four studies investigating SkQ1 in rat models of AD were identified, and all demonstrated favourable neuroprotective effects to varying degrees of benefit. The earliest study investigating the effects of SkQ1 (Plastoquinone decyltriphenylphosphonium) examined the effect of a single injection of the compound into healthy male wistar rats, followed by incubation of their brain tissue with Aβ peptide [32]. The study concluded that SkQ1 was able to abolish Aβ’s inhibition of LTP in the hippocampus, postulating that this could improve cognition in vivo, potentially contributing towards an effective treatment of AD.

Multiple in vivo studies comparing the OXYS rat model of AD to healthy control rats were included from the research groups of Stefanova et al. [33,34] and Kolosova et al. [35] in Russia. These groups first tested long-term treatment with SkQ1 in young OXYS rats to represent the human pre-clinical stage through to active disease of AD [33]. In this group, SkQ1 decreased Aβ and p-tau concentrations in OXYS rat hippocampi and cortex, improved age-related behavioural deficits by 13 months old, improved locomotor activity by 3 months old and showed a non-significant trend towards improving spatial learning and memory in 13-month-old OXYS rats [33]. The group then executed a similar study with SkQ1 in OXYS rats in the active disease stage of AD, finding improved mitochondrial structure and markers of mitochondrial function and biogenesis, such as increased enzymatic activity of complex IV in the ETC, increased neurons in the hippocampus and neurotrophic supply, improved synaptic structure, density and function and restored hippocampal Aβ and p-tau levels to controls. This contributed to observed improvements in hippocampus-dependent learning and memory in OXYS rats treated with SkQ1 [34]. Finally, the same group explored SkQ1’s role in aged OXYS rats to represent end-stage AD, exhibiting reduced Aβ burden in the hippocampus, improved mitochondrial structure and some improved aspects of mitochondrial biogenesis. Consequently, restoration of some locomotor and exploratory activities was observed in these rats, indicating possible benefit in end-stage disease [35].

## 4. Discussion

Disease-modifying drugs have recently been employed to treat AD. However, their benefits are short-term, and they cannot be used to treat other forms of dementia. We were the first to report that oxidative stress was an important feature of AD [51]. Since then, considerable evidence has been provided to demonstrate that oxidative stress is central to the pathogenesis not only of AD but all forms of dementia, arguing strongly for investigating the role of antioxidants to prevent and treat AD and other neurodegenerative diseases.

This scoping review summarised in vivo evidence from animal studies on the effects of NVAs and MTAs in mitigating MCI, AD and other forms of dementia. Across the included studies, a range of these compounds demonstrated varying degrees of neuroprotective effects through antioxidant, mitochondria-protective and/or cognition-enhancing features in diverse animal models of cognitive dysfunction, with AD animal models being the most researched.

### 4.1. Natural Vitamin Antioxidants

Vit.E is a well-established antioxidant and its ability to prevent or treat diseases involving oxidative stress has been widely researched [52,53]. Consistent with this, Vit.E was found to decrease markers of oxidative stress in all in vivo studies included in this review and improve cognitive outcomes following Vit. E, Trolox or α-tocopherol administration [25,27,28]. Other included NVAs—methylcobalamin, folate, niacin, thiamine, pantetheine and retinoic acid—also displayed varying degrees of overall positive effects on oxidative stress and/or cognition via mechanisms including transcription factor and epigenetic modulation, mitochondrial biogenesis and redox homeostasis.

Despite these promising preclinical findings, these benefits have not been consistently reproducible in human trials of AD and other dementias, with many reasons postulated for this discrepancy [53,54,55,56,57]. Proposed explanations include limitations in study design, such as small sample size, short intervention period, suboptimal dose translation to humans, and heterogeneity in disease stage at enrolment [55,56]. Additionally, limited brain bioavailability of NVAs due to restricted BBB impermeability may reduce therapeutic efficacy in humans [55,58]. This limitation underscores the need for targeted antioxidant therapies such as MTAs to hopefully address the current failure of antioxidant therapy in human trials. Furthermore, whilst B vitamins possess antioxidant properties, their primary proposed mechanism in mitigating cognitive decline is via lowering Hcy levels [59]. This may partly explain the inconclusive evidence for B vitamin supplementation in human cognitive decline and dementia, targeting Hcy as opposed to oxidative stress, which is central to the pathophysiology of AD and cognitive dysfunction [60].

### 4.2. Mitochondria-Targeted Antioxidants

The MTAs included in this review exhibited consistently promising effects on cognitive function, oxidative stress and mitochondrial function. The peptide SS31 was the most extensively studied, closely followed by the TTP (triphenylphosphonium)—conjugated antioxidants SkQ1, MitoQ and MitoTEMPO. All in vivo trials of SS31 showed strong antioxidant effects, enhanced mitochondrial function, and improved cognition in mice [36,37,38,39,40]. SS31 is a tetrapeptide that penetrates the MM where it associates with cardiolipin and exerts antioxidant effects [49]. Cardiolipin is a crucial component of the inner MM and the most sensitive component to ROS, leading to downstream inactivation of ETC components, mitochondrial structure disruption and eventually apoptosis; therefore, it is a very important target of SS31, potentially facilitating the robust effects in these studies [45,61].

The in vivo studies investigating SkQ1 were predominantly performed in rat models of AD across early to late disease stages by the same research group in Russia, and all demonstrated improvements in mitochondrial and cognitive outcomes [33,34,35]. Remarkably, oxidative stress was not elevated in the OXYS rat model of AD; however, SkQ1 was able to reduce or eliminate many aspects of ageing, primarily through targeting mitochondrial structure and function [62]. This may indicate alternate therapeutic pathways for MTAs involving mitochondrial biogenesis besides their antioxidant capabilities. Furthermore, SkQ1’s antioxidant effects have been well documented in other preclinical studies, therefore suggesting that the OXYS model may not sufficiently mimic the oxidative stress model of AD [45].

MitoQ improved cognition and pathological outcomes in AD models without significant alterations in oxidative stress markers or mitochondrial DNA, instead acting through preservation of ETC elements. This observed improvement in disease pathogenesis without altering oxidative stress, combined with the structural similarity between MitoQ and SkQ1, which only differ in the ubiquinone versus plastoquinone moiety as the antioxidant further supports alternative mitochondrial-modulating mechanisms beyond ROS scavenging in MTAs [45].

Collectively, these findings support the therapeutic potential of novel MTAs in preventing and/or treating cognitive decline and dementia by directly targeting mitochondrial redox balance and biogenesis in vivo. In particular, SS31 and SkQ1 demonstrated more consistent, reproducible and robust benefits across a range of cognitive and cellular domains in various study designs reviewed. Furthermore, MTAs have shown favourable safety and efficacy profiles in human trials of various other non-neurological diseases such as dry eye syndrome (SkQ1), vascular disease and chronic liver disease (MitoQ), and primary mitochondrial myopathy, heart failure and ischemic injury (SS31) [61,63]. However, to date, no human clinical trials have evaluated MTAs in dementia and/or MCI populations

### 4.3. Comparative Outcomes and Clinical Relevance

Both NVAs and MTAs improved oxidative and mitochondrial outcomes across the in vivo studies reviewed, although their consistency and translational maturity differ. NVAs such as Vitamins E, C, A and B-complex have been evaluated in human trials of cognitive decline, yielding mixed or marginal effects, likely limited by short half-life, poor blood–brain barrier penetration and off-target metabolism. In contrast, MTAs including MitoQ, MitoTEMPO, SS-31 and SkQ1 showed more reproducible benefits in preclinical models, improving mitochondrial integrity, synaptic function and cognition, but have yet to be formally tested for cognitive outcomes in humans. Their ability to accumulate within mitochondria addresses key pharmacokinetic constraints of conventional antioxidants, and existing clinical safety data from non-neurological indications support advancement to early-phase trials in mild cognitive impairment and dementia. Direct comparative studies will be needed to establish the relative efficacy and therapeutic potential of MTAs versus established antioxidant strategies.

### 4.4. Strengths and Limitations

This review comprehensively assessed current in vivo evidence examining the effects of MTAs and NVAs on oxidative stress, mitochondrial function and cognitive outcomes, capturing a wide range of antioxidant compounds. The results of these studies were consistently beneficial in at least one of these domains, with MTAs showing the most consistent improvements to in vivo animal models of dementia or cognitive decline. To the best of our knowledge, this is one of the first reviews to systematically evaluate both NVAs and MTAs, specifically in the context of in vivo animal models of AD and other forms of dementia, and it provides a more comprehensive and up-to-date overview of NVAs and MTAs in cognitive health compared to other reviews.

A key limitation is the heterogeneity of different animal models, pathological targets, and intervention timing, which complicates cross-study comparisons and limits translational inference to human cognitive decline and dementia. A further limitation of the established animal models is their generation using transgenes, such as the APP, PS1 and PS2 gene mutations, which are more reflective of hereditary AD in humans; however, this contributes to <5% of all total AD in humans [64,65]. Accordingly, some models in this review utilised accelerated ageing models, such as the SAMP8 mouse and OXYS rat models, to better reflect the sporadic development and multi-faceted nature of AD in humans, with these models also having promising results in the included studies [65]. Furthermore, some models examined cognitive impairment secondary to environmental or metabolic factors such as pollutants or hyperglycaemia, which may not accurately capture the pathophysiological processes underlying age-related or primary neurodegenerative cognitive decline in humans. Finally, whilst MTAs such as MitoQ and SkQ1 demonstrated exclusively antioxidant and positive effects in the included studies, prior evidence indicates that they may exert pro-oxidative effects under certain conditions, with MitoQ exhibiting a narrower window of pro- to antioxidant concentrations than SkQ1 [66,67]. Therefore, further research is needed to clarify these contexts to ensure safety, with studies in primate models and ultimately well-designed human trials. Finally, despite the promising preclinical results, translatability from experimental models to clinical benefit is not guaranteed, due to inter-individual variability in transporter expression, metabolism and mitochondrial uptake of natural vitamin antioxidants and mitochondria-targeted antioxidants, which remains inadequately characterised in humans and may influence brain exposure, dosing and therapeutic efficacy but are rarely considered in preclinical or early clinical studies.

### 4.5. Implications and Future Recommendations

The promising findings of this review highlight the potential of MTAs as therapeutic candidates for cognitive decline and dementia. Future research should investigate their efficacy across various subtypes of dementia and disease stages, while also determining optimal dosing, timing of intervention and their long-term safety. Given that the in vivo studies in this review were largely undertaken in rodent models, an important next step would be to evaluate the most promising candidates in primate models to determine likely translation to human. Nevertheless, considering their favourable safety profiles in other clinical populations, early-phase human trials may be worth undertaking to translate these promising preclinical findings into effective clinical application for dementia and other neurocognitive disorders, for which disease-modifying therapies remain limited.

## 5. Conclusions

This scoping review provides the first integrated synthesis of in vivo evidence on NVAs and novel MTAs in cognitive decline and dementia, identifying key translational gaps and future research directions. While both antioxidant classes exhibited neuroprotective effects, MTAs demonstrated more consistent improvements in oxidative stress, mitochondrial function and cognition compared with NVAs, likely reflecting their better targeting of mitochondrial redox balance and biogenesis to exert their antioxidant effect. Early-phase human clinical trials of MTAs in dementia and MCI are urgently warranted following initial validation in primate studies to determine their efficacy in slowing or preventing disease progression.

## Figures and Tables

**Table 1:** Summary of study characteristics and outcomes of included in vivo studies.

Title	Author/s	Year	Country	Aims	Population (Tx)	Tx, Dose, Duration	Methods	Primary Outcomes	Results
Benfotiamine treatment activates the Nrf2/ARE pathway and is neuroprotective in a transgenic mouse model of tauopathy [16].	Tapias et al. [16]	2018	USA	To investigate whether benfotiamine prevents the formation of NFTs and confers neuroprotection in a mouse model of tauopathy.	P301S TG mice 1 months (F:M 1:1)	Benfotiamine, 200 mg/kg PO, 9 mo	Mice were treated as described from 1 month of age and cognitively tested at 5, 7 and 9 months of age. They were sacrificed at 10 months of age for histopathological analysis.	p-tau, NFTs, ROS, cognitive function, lifespan	Benfotiamine reduced NFTs, was neuroprotective and reduced mitochondrial dysfunction, resulting in improved cognition and lifespan in a mouse model of tauopathy.
The role of folate receptor alpha in the partial rejuvenation of dentate gyrus cells. Improvement of cognitive function in elderly mice [17].	Antón-Fernández et al. [17]	2024	Spain	To explore whether aged mice brain cells can be rejuvenated through use of methyl donors, folate and methionine.	C57BL/6 wild-type mice 21 months (M *n *= 1, F *n *= 6)	Folate, 0.25 mg/mL, Once	Treatment as listed was injected into the DG of mice brain hemispheres. Mice then underwent cognitive testing, sacrifice and extraction of brain tissue.	Folate cellular effects, cognitive function	Infusion with folate in aged mice was able to improve some aspects of cognition and rejuvenate DG cells, independent of sex.
Folic acid and S-adenosylmethionine reverse Homocysteine-induced Alzheimer’s disease-like pathological changes in rat hippocampus by modulating PS1 and PP2A methylation levels [18].	Sun et al. [18]	2024	China	To elucidate the mechanism by which elevated homocysteine levels cause AD-like pathology and whether folic acid and S-adenosylmethionine improve this in vitro and in vivo.	Wistar rats (M, *n *= 32)	FA, 2 mg/kg, IP, 4 wks	Adult male rats were treated with IP Hcy +/− FA as described, compared to controls then sacrificed for plasma and hippocampal analyses.	Hcy, Aβ, p-tau	Rats exposed to Hcy and subsequently treated with FA showed decreased plasma Hcy. Rat hippocampal neurons showed decreased burden and markers of both Aβ and p-tau.
Methylcobalamin alleviates neuronal apoptosis and cognitive decline induced by PM2.5 exposure in mice [19].	Ji et al. [19]	2022	China	To explore whether Vitamin B12 is neuroprotective against cognitive impairment and apoptosis induced by chronic PM2.5 exposure.	C57BL/6 mice, 6 wk (M, *n* = 8)	MeCbl 1.25 mg/L, PO, 6 mo	Mice were exposed to either filtered air or PM2.5 12 h per day, 6 days per week and treated with MeCbl compared to negative controls. They were then subjected to cognitive testing and sacrificed for brain tissue and blood analysis.	ROS, neuronal apoptosis, cognitive function	MeCbl treatment in mice was able to reduce PM2.5 induced neuronal apoptosis, via improving markers of oxidative stress and mitochondrial function, resulting in improved cognition.
Nicotinamide riboside restores cognition through an upregulation of proliferator-activated receptor-γ coactivator 1α-regulated β-secretase 1 degradation and mitochondrial gene expression in Alzheimer’s mouse models [20].	Gong et al. [20]	2013	USA	To investigate if NR treatment in an AD mouse model could attenuate Aβ toxicity through activation of PGC-1a-mediated BACE1 degradation.	PGC-1α/Tg2576 mice, 5–6 months	NR, 250 mg/kg PO, 3 mo	Mice were treated, compared to controls, then subjected to cognitive testing and sacrificed for brain tissue analysis.	Aβ, LTP, PGC-1α, BACE1, cognitive function,	NR attenuated Aβ toxicity, improved synaptic plasticity, abolished reduced LTP and improved cognitive function in a mouse model of AD.
Nicotinamide riboside modulates the reactive species interactome, bioenergetic status and proteomic landscape in a brain-region-specific manner [21].	Marmolejo-Garza et al. [21]	2024	The Netherlands	To investigate the effects of nicotinamide riboside (NR) on apoptosis, inflammation and protein expression for both in vitro and in vivo models of AD.	3xPB mice, 16–18 months	NR, 12 mM, 8 wks	Mice were treated as described and compared to controls. They were then sacrificed and brain tissue was analysed.	ROS, neuronal apoptosis, RSI, proteome	NR treatment reduced oxidative stress, apoptosis and some proteomic changes in an AD mouse model.
Long-term pantetheine treatment counteracts pathologic gene dysregulation and decreases Alzheimer’s disease pathogenesis in a transgenic mouse model [22].	Baranger et al. [22]	2019	France	To investigate the potential in vivo benefits of pantetheine supplementation in a mouse model of AD.	5XFAD mice 1.5 months (*n *= 10)	Pantetheine 15 mg, IP, 3x/wk 5.5 mo	Mice were treated as described at 1.5 months of age, subjected to behavioural testing at 7 months, then sacrificed for brain histopathological analysis.	Aβ, astrogliosis, microgliosis, behaviour	Pantetheine supplementation in a mouse model reversed AD induced astrogliosis, microgliosis, Aβ plaque burden and aggressive behaviour.
Alleviation of a selective age-related relational memory deficit in mice by pharmacologically induced normalisation of brain retinoid signalling [23].	Etchamendy et al. [23]	2001	France	To determine the effect of ageing on LTP in the brain of aged mice and whether RA has a promnesic effect.	Male mice C57BL/6 Jico strain 21–23 months	RA (150 ug/kg) (*n* = 10) or RA + CD3106 (*n* = 6)	Aged or control mice were injected w RA as described with or without RAR antagonist before undergoing cognitive testing followed by sacrifice and brain tissue analysis.	LTP, cognitive function, brain RAR expression	RA treatment partially improved LTP, increased brain expression of RAR and improved cognitive function in aged mice.
All-trans retinoic acid improved impaired proliferation of neural stem cells and suppressed microglial activation in the hippocampus in an Alzheimer’s mouse model [24].	Takamura et al. [24]	2017	Japan	To determine whether the suppression of NSC proliferation in a mouse model of AD is impacted by ATRA (All-trans retinoic acid).	3xTg AD mice 12 months (F)	ATRA, 20 mg/kg, IP 3x/wk for 4 wks	Mice were treated as described with ATRA or control, then sacrificed and brain tissue extracted for histological analysis.	NSC proliferation, microglial and astrocyte activation	ATRA improved NSC proliferation and reduced neuroinflammation in a mouse model of AD.
Long-term antioxidant supplementation attenuates oxidative stress markers and cognitive deficits in senescent-accelerated OXYS rats [25].	Kolosova et al. [25]	2006	Russia	To verify the correlation between brain ageing and oxidative stress and investigate the effect of long-term Vitamin E or flavonoid supplementation in preventing cognitive deficits.	OXYs + Wistar rats, 2 months (M)	α-tocopherol, 30 mg/kg, 4 courses	Rats were treated in 4 courses as described in 2 month increments with either Vitamin E or a flavonoid starting at 3 mo old and compared to controls. Cognitive testing was undertaken, and rats were sacrificed for brain and serum analyses.	ROS, cognitive function	α-tocopherol treated OXYS rats had improved short-term memory and decreased markers of oxidative stress.
Plaque-derived oxidative stress mediates distorted neurite trajectories in the Alzheimer mouse model [26].	Garcia-Alloza et al. [26]	2006	USA	To determine if antioxidants Vitamin E and ginko biloba diminish reactive oxygen species in a mouse model of AD.	APPswe/PS1d9 mice 6–10 months	Trolox, 210 mg/kg, 24 h/15 d	Mice in the acute treatment group received Trolox prior to cortical surgery, then were imaged. The chronic treatment group were treated as described for 15 days, sacrificed then imaged.	Aβ, ROS, neuronal morphology	Trolox treatment decreased Aβ plaque-related oxidative stress and dystrophic neurites in a mouse model AD.
PM2.5 induced neurodegenerative-like changes in mice and the antagonistic effects of Vitamin E [27].	Liu et al. [27]	2019	China	To investigate the effects of PM2.5 exposure on the brain and whether Vitamin E can ameliorate the pathology.	C57BL/6J mice, 6 wk (*n *= 10)	Vitamin E, 50 mg/kg, 2 wks	Mice were exposed to PM2.5 for 1 week and treated as described with concurrent Vitamin E or control, sacrificed and brain tissue was analysed.	Neurodegeneration, cognitive function	Mice exposed to PM2.5 recorded cognitive deficits and increased markers of neurodegeneration, which was blocked by Vitamin E treatment.
Suppression of tau-induced phenotypes by Vitamin E demonstrates the dissociation of oxidative stress and phosphorylation in mechanisms of tau toxicity [28].	Cowan et al. [28]	2021	UK	To investigate if Tau protein induces pathological changes in vivo and if these changes are rescued by treatment with Vitamin C or E.	Tg Tau drosphila melanogaster (*n* = 30/group)	Vitamin E: 0.5, 1.5 or 4.5 mM, Vitamin C 100 uM or 10 mM	Flies were given food with listed concentrations of vitamins and their motor activity was analysed. Once they reached adulthood, they were sacrificed for brain tissue, with a small subgroup undergoing further testing.	Tau, ROS, neuronal function	Treatment with either Vitamin E or C leads to reduced oxidative damage, improved neuronal function and improved behaviour in a d melanogaster model of tauopathy.
The mitochondria-targeted antioxidant MitoQ prevents loss of spatial memory retention and early neuropathology in a transgenic mouse model of Alzheimer’s disease [29].	McManus et al. [29]	2011	USA, UK	To examine the ability of MitoQ to prevent AD like pathology in mouse cortical neurons and a mouse model of AD.	3xTg-AD mice 2 months (F, *n* = 117)	MitoQ, 100 uM, PO, 5 mo	Mice were treated with MitoQ, compared with negative controls. They were then subjected to cognitive testing, followed by sacrifice and analysis of brain tissue.	Aβ burden and neurotoxicity, ROS, mitochondrial function, cognitive function	MitoQ treatment reduced oxidative stress, synaptic loss and astrogliosis in the cortex and decreased Aβ burden in the hippocampus and neocortex. This restored cognitive performance to the level of WT mice.
The mitochondria-targeted antioxidant MitoQ extends lifespan and improves healthspan of a transgenic Caenorhabditis elegans model of Alzheimer disease [30].	Ng et al. [30]	2014	Singapore	To examine the efficacy of MitoQ in reducing Aβ induced pathology and oxidative stress in a *C. elegans* model of AD.	Tg *C. elegans* CL2006 (*n* = 200)	MitoQ, 0.1, 1, 5 µM, 2 d	Treatment was administered as described and compared to controls, with concurrent behaviour analysis, followed by post-treatment tissue analysis.	Aβ toxicity, mitochondrial function, lifespan and behaviour	MitoQ treatment prolonged the lifespan and healthspan in a *C. elegans* AD model, mediated through its interaction with complex I and IV of the ETC.
Mito-TEMPO, a mitochondria-targeted antioxidant, improves cognitive dysfunction due to hypoglycemia: an association with reduced pericyte loss and blood–brain barrier leakage [31].	Lin et al. [31]	2022	China	To investigate the mechanism of hypoglycaemia induced cognitive dysfunction and whether this is reversed by MitoTEMPO in vivo.	C57BL/6J mice (M, *n* = 100)	MitoTEMPO, 0.7/mg/kg, 10 d	Mice were induced to hypoglycaemia then treated as described, compared to controls, and either sacrificed for histological testing or subjected to cognitive testing.	BBB leakage, ROS, pericyte loss and apoptosis, mitochondrial function	MitoTEMPO reduced oxidative stress, pericyte loss and apoptosis, protected against BBB leakage and neuron damage and this led to improved cognition in a mouse model of cognitive dysfunction.
Mitochondria-targeted plastoquinone antioxidant SkQ1 prevents amyloid—induced impairment of long-term potentiation in rat hippocampal slices [32].	Kapay et al. [32]	2013	Russia	To investigate whether SkQ1 attenuates Aβ -induced impairment of LTP in rat hippocampi.	Wistar rats (M)	SkQ1 250 nmol/kg, IP, Once	Rats were injected with a single dose of SkQ1 as described, compared with controls, and sacrificed 24 h later for incubation with Aβ and subsequent hippocampal tissue analysis.	Aβ, LTP	SkQ1 treatment in rats rescues the inhibitory effect of Aβ peptide on LTP induction in hippocampus.
Alzheimer’s disease-like pathology in senescence-accelerated OXYS Rats can be partially retarded with mitochondria-targeted antioxidant SkQ1 [33].	Stefanova et al. [33]	2014	Russia	To study the influence of long-term treatment with SkQ1on the OXYS rat model of AD.	OXYS rats 1.5 months (M, *n *= 15)	SkQ1, 250 nmol/kg PO, 21.5 mo	Rats were treated as described and compared to healthy controls, subjected to behavioural testing at multiple time points then sacrificed at 23 mo for brain tissue analysis.	Aβ, p-tau, cognitive function	Long-term SkQ1 treatment in the OXYs model of AD restored some domains of cognitive function and reduced Aβ and p-tau burden.
An antioxidant specifically targeting mitochondria delays progression of Alzheimer’s disease-like pathology [34].	Stefanova et al. [34]	2016	Russia	To determine the role of mitochondrial damage in AD and whether SkQ1 can alleviate this.	OXYS Rats 12 months (M, *n *= 15)	SkQ1, 250 nmol/kg PO, 6 mo	Rats were treated as described, subjected to behavioural testing then sacrificed for brain histopathological analysis.	Aβ, p-tau, mitochondrial function, neuronal cell function, cognitive function	SkQ1 treatment in rats restores mitochondrial function, resulting in increased neurons and synapses, decrease in Aβ and tau burden and improved cognition.
Antioxidant SkQ1 alleviates signs of Alzheimer’s disease-like pathology in old OXYS rats by reversing mitochondrial deterioration [35].	Kolosova et al. [35]	2017	Russia	To evaluate the efficacy of SkQ1 in an aged AD rat model in mitigating significant AD-induced pathology.	OXYS rats 19 months (M, *n *= 15)	SkQ1, 250 nmol/kg PO, 5 mo	Rats were treated as described and compared to healthy controls, subjected to behavioural testing or sacrificed for brain tissue analysis at 24 mo old.	Aβ, p-tau, mitochondrial function, cognitive function	Treatment with SkQ1 in an aged OXYS rat model of AD resulted in some improved markers of mitochondrial function, Aβ burden and certain behavioural alterations.
SS31, a small molecule antioxidant peptide, attenuates β-Amyloid elevation, mitochondrial/synaptic deterioration and cognitive deficit in SAMP8 mice [36].	Jia et al. [36]	2016	China	To assess mitochondrial and synaptic alterations in a mouse model of AD and whether SS31 can mitigate these and improved cognition.	SAMP8 mice 10 months (M, *n *= 10)	SS31, 5 mg/kg IP, 8 wks	Mice were treated as described compared to negative controls. They were subjected to behavioural testing after 8 weeks then sacrificed for brain tissue analysis.	Aβ, mitochondrial and synaptic function, cognitive function	SS31 treatment reduced hippocampal Aβ burden, protected mitochondrial dynamics and function, preserved synaptic function and improved cognitive performance in a mouse model of AD.
Mitochondria-targeted small molecule SS31: a potential candidate for the treatment of Alzheimer’s disease [37].	Reddy et al. [37]	2017	USA	To improve understanding of the protective effects of SS31 in AD-associated mitochondrial and synaptic dysfunction in a mouse model of AD.	APP Tg2576 mice 12 months (*n *= 5)	SS31 5 mg/kg IP, 6 wks	Mice were treated as described and compared to negative controls. They were sacrificed for brain and plasma analysis at 6 weeks.	Aβ, mitochondrial and synaptic function	SS31 lowered markers of AD pathology such as Aβ production, mitochondrial dysfunction and impaired biogenesis and synaptic dysfunction in a mouse model of AD.
Elamipretide (SS-31) improves mitochondrial dysfunction, synaptic and memory impairment induced by lipopolysaccharide in mice [38].	Zhao et al. [38]	2019	China	To assess neuroprotective effects of SS-31 against LPS-induced oxidative stress and cognitive dysfunction.	C57BL/6 mice 10–11 wk (M, *n *= 96)	SS31, 5 mg/lg, 4 d	Mice were induced to oxidative stress by LPS, treated as described, then subjected to various cognitive tests compared to controls.	ROS, neuroinflammation, mitochondrial function, cognitive function	SS31 protected against mitochondrial dysfunction, oxidative stress, synaptic dysfunction and regulated signalling pathways in this mouse model of cognitive impairment, leading to reduced learning and memory deficits.
The mitochondria-targeted small molecule SS31 delays progression of behavioural deficits by attenuating b-amyloid plaque formation and mitochondrial/synaptic deterioration in APP/PS1 mice [39].	Jia et al. [39]	2023	China	To examine the effect of SS31 on a disease progression in an early mouse model of AD.	APP/PS1 Tg mice 8 months (M, *n *= 12)	SS31, 3 mg/kg IP, 3x/wk for 24 wks	The APP/PS1 mouse model was compared to control mice and treated as described. Behaviour was tested at 22 weeks, then mice were sacrificed at 24 weeks.	Aβ, ROS, mitochondrial and synaptic function, cognitive function	SS31 lowered markers of AD pathology such as apoptosis, Aβ burden, ROS, mitochondrial dysfunction and improved cognitive and behavioural deficits in a mouse model of AD.
Real-time imaging of mitochondrial redox reveals increased mitochondrial oxidative stress associated with amyloid β aggregates in vivo in a mouse model of Alzheimer’s disease [40].	Calvo-Rodriguez et al. [40]	2024	USA	To visualise the effects of Aβ on mitochondrial oxidation in vivo and determine whether this can be attenuated using SS-31.	APP/PS1 Tg mice 8 months (M, F *n* = 3)	SS31, 5 mg/kg, 2x/wk for 8 wks	Mice were treated as described compared to controls. After treatment mice were imaged in real time using multiphoton microscopy and then sacrificed for brain tissue analysis.	ROS, Aβ, neurite morphology	Mitochondrial oxidative stress in a mouse model of AD imaged in real time was inhibited by treatment with SS31 and Aβ plaque- associated dystrophic neurites were decreased without any observed decrease in Aβ plaque burden.

Aβ (Amyloid Beta), AD (Alzheimer’s disease), APP (amyloid precursor protein), ATRA (All-trans retinoic acid), BACE1 (β-secretase), *C. elegans* (Caenorhabditis elegans), CD3106 (RAR antagonist), DG (dentate gyrus), ETC (electron transport chain), FA (folic acid), IP (intraperitoneal), LPS (lipopolysaccharide) LTP (long-term potentiation), MeCbl (methylcobalamin), NFTs (neurofibrillary tangles), NR (nicotinamide riboside), NSC (neural stem cell), p-Tau (Phosphorylated Tau), PO (Per Oral), RA (retinoic acid), RAR (retinoic acid receptor), ROS (reactive oxygen species), RSI (reactive species interactome), SS-31 (Elamipretide), Tg (transgenic), UK (United Kingdom), USA (United States of America), WT (wild type), wks (weeks).

**Table 2:** The mechanisms of action of novel MTAs included in the review.

MTA	Mechanisms of Action
MitoQ	The antioxidant Ubiquinone is conjugated to TPP, a lipophilic cation which passes through the lipid bilayer and accumulates in mitochondria due to its electrostatic potential with the mitochondrial membrane. Ubiquinone is reduced primarily by complex II of the ETC into its active form ubiquinol which exerts its antioxidant effect by scavenging ROS and targeting lipid peroxidation [44].
SkQ1	The antioxidant plastoquinone, a component of the chloroplast ETC-containing methyl groups in place of methoxy groups in ubiquinone, is conjugated to TPP. It accumulates in mitochondria in a similar fashion to MitoQ and once reduced to plastoquinol exerts its antioxidant effect, also scavenging ROS and targeting lipid peroxidation [45,46].
MitoTEMPO	The antioxidant TEMPO is conjugated with TPP, again accumulating in mitochondria via the same mechanism as above [47]. TEMPO is a synthetic superoxide scavenger which acts as a SOD mimic, neutralising ROS and sustaining redox equilibrium [48].
SS31 (Elamipretide)	The Szeto Schiller peptides are small hydrophilic aromatic–cationic tetrapeptides which penetrate cell membranes and associate with cardiolipin in the inner mitochondrial membrane, stabilising its structure [49]. Its antioxidant effects include inhibiting ROS generation and lipid peroxidation, and mitochondria-protective effects include preventing cytochrome C release and inhibiting MPT to prevent cell swelling and death [50].

ETC (electron transport chain), MitoQ (Mitoquinone Mesylate), MitoTEMPO (TPP- Conjugated TEMPO) MPT (mitochondrial permeability transition), ROS (reactive oxygen species), SkQ1 (Plastoquinone-decyltriphenylphosphonium), SOD (superoxide dismutase), SS-31 (Szeto- Schiller 31), TEMPO (2,2,6,6-Tetramethylpiperidine 1-oxyl), TPP (triphenylphosphonium).

## Data Availability

The original contributions presented in this study are included in the article/Supplementary Materials. Further inquiries can be directed to the corresponding author(s).

## Supplementary Materials

The following supporting information can be downloaded at: https://www.mdpi.com/article/10.3390/antiox15010078/s1, Table S1: EMBASE Search; Table S2: PRISMA-Scr Checklist.
